# A Participatory Curricula for Community Health Workers and Supervisors to Increase HIV Health Outcomes

**DOI:** 10.3389/fpubh.2021.689798

**Published:** 2021-07-30

**Authors:** Serena Rajabiun, Allyson Baughman, Marena Sullivan, Beth Poteet, Alicia Downes, Jo Ann Whitlock Davich, Simone Phillips, Precious Jackson, LaTrischa Miles, Mari-Lynn Drainoni, Emmitt Maurice Evans, Sara S. Bachman, Linda Sprague Martinez

**Affiliations:** ^1^Department of Public Health, University of Massachusetts, Lowell, MA, United States; ^2^Center for Innovation in Social Work & Health, Boston University School of Social Work, Boston, MA, United States; ^3^Multnomah County Health Department, Portland, OR, United States; ^4^AIDS United, Washington, DC, United States; ^5^Stokely Phillips Griffin Group LLC, St Louis, MO, United States; ^6^Pasadena Public Health Department, Consultant, Pasadena, CA, United States; ^7^KC Care Health Center, Consultant, Kansas City, MO, United States; ^8^Section of Infectious Diseases, Boston University School of Medicine, Boston, MA, United States; ^9^Department of Health Law, Policy & Management, Boston University School of Public Health, Boston, MA, United States; ^10^Evans Center for Implementation and Improvement Sciences, Boston University, Boston, MA, United States; ^11^School of Social Policy & Practice, University of Pennsylvania, Philadelphia, PA, United States

**Keywords:** community health workers, training, supervision, capacity building, HIV Education

## Abstract

Community Health Workers (CHWs) are becoming essential members of the HIV workforce as emerging evidence demonstrates their effectiveness in engaging people with HIV into care and treatment. In 2018, among the estimated 37,000 persons who received an HIV diagnosis, the majority were from racial ethnic minority communities. CHWs serve as a bridge between the community and health care system and have the potential to address structural inequities and reduce the stigma, discrimination and other barriers that prevent people with HIV from seeking and staying in care and treatment. Effective CHW integration into the HIV primary care team requires a training and supervision system that is culturally responsive to the complex social and medical needs of people with HIV. This article describes a comprehensive training approach and curricula for CHWs and supervisors and its impact on the health care team. Grounded in a Popular Education model and using the CHW core consensus competency (C3) framework, a team of experts in HIV, training and supervision, including CHWs working in HIV care and treatment developed an 80-h CHW and 20-h supervisor curricula. The trainings were delivered via in-person and virtual sessions over the course of 2 years. Using a mixed method evaluation, 23 CHWs and 22 supervisors across 10 clinic sites in eight states participated in the training sessions. Measures included knowledge and confidence related to HIV-specific content, supporting clients with managing stigma and discrimination, ability to communicate with other team members and helping clients navigate the services system. CHWs reported improved skills with documentation in the electronic health record, helping clients with treatment adherence challenges and educating on lab results. Supervisors reported learning strategies for assigning clients to CHWs, self-care techniques, providing strengths-based feedback, and mentoring and coaching. The participatory practice-based curricula allowed supervisors and CHWs to share experiences and solicit input from peers for problem resolution and implementation of new policies and practices. This training approach focused on HIV specific content with core competency training could serve as a model for CHWs working in primary care settings and with populations experiencing multiple chronic health conditions and social needs.

## Introduction

An estimated 1.2 million people are living with HIV in the United States, with racial/ethnic minority communities experiencing the greatest burden. In 2018, among the 37,000 new diagnoses, 47% were from Black/African American communities and 23% were Hispanic/Latinx. (1) Despite new advances in treatment, only 61% of people with HIV are virally suppressed. To address these inequities and reduce new infections, the National Plan to End the HIV Epidemic and the recently updated National HIV Strategy focus on using community-based interventions to reduce new infections among people at risk for HIV and expanding treatment access for people with HIV by 2025. (2) Community Health Workers (CHWs) can play an integral role in reaching those goals. There is emerging evidence that CHWs, including patient navigators and peer educators, as members of the HIV workforce can effectively link and retain people with HIV in care and improve viral suppression. (3, 4) By functioning as a bridge between the community and health care system, CHWs have the potential to address the structural inequities and reduce the stigma, discrimination and other barriers that prevent people at risk for and living with HIV from seeking and staying in care and adhering to treatment. (5) Though prior studies demonstrate the potential of CHW support services to improve HIV outcomes, few resources exist to guide the process of integrating CHWs into care teams in a clinical setting.

Effective integration of CHWs into the HIV primary care team requires a training and supervision system that is culturally responsive to the complex social and medical needs of the HIV population. (6–13) Challenges to integration are attributed to lack of clearly defined CHW roles as member of a care team, limited reimbursement by insurers, and limited or no access to resources to gain knowledge and enhance skills to carry out their services unlike other health professional programs. (14–18) There are CHW certification programs in the US which can enhance the credibility of the CHW role. (19, 20) New training programs such as ECHO, a distance learning program that has been applied to obesity prevention and addiction management training, offer promising models and approaches specifically for continuing education in specialty areas. (16, 17) Current studies on CHW training have emphasized the importance of competency-based training programs tailored to skills, workplace settings, and CHW scope of practices (21–23).

In HIV care, there is limited information about standardized training curricula and programs for CHWs as part of the HIV health care workforce. National HIV training programs have focused on the clinical care workforce, rather than non-clinical support staff such as CHWs, using on-line sessions to improve HIV knowledge and updates on treatment as well as preceptorships and mentoring programs for managing and treating patients with HIV. (24) While there is no literature focused on general CHW training in HIV care, there are limited studies focused on training programs for developing a system of peer educators. Previous studies found the importance of strengthening the knowledge and skills of people with HIV to serve as peer educators in the HIV clinical team. Key elements for these training programs include building competencies on HIV knowledge and treatment, identifying the peer roles in the HIV care team and enhancing communication skills. (25) Lessons learned from these national training programs also point to the need for continued education on topics and financial support for training and instruction about how to include peers as part of a training and education team.

In 2016, the Health Resources and Services Administration funded a 3 year project: *Improving Access to Care: Using Community Health Workers to Improve Linkage and Retention in HIV Care*. (26) The project provided training, technical assistance and funding to 10 Ryan White-HIV/AIDS Program funded agencies to enhance the integration of CHWs into the care team. The sites were located in eight states across the US. Seven sites served a predominantly urban population while three served a predominantly rural catchment area. Details of the funded sites are published elsewhere. (3) This article describes the national, comprehensive training approach and curricula for CHWs and their supervisors delivered to support effective integration of CHWs into the HIV health care team. Results and lessons learned on CHW and supervisors skills and impact on the health care team are provided. The findings and recommendations provide implementation strategies and mechanisms to enhance CHW efforts as part of the HIV and primary care workforce.

## Pedagogical Framework and Methods

The pedagogical framework for the training program was grounded in Popular Education (26) and the Community Health Worker Core Consensus (C3) Project's core competencies for CHWs. Popular Education, a social movement founded by Brazilian educator and philosopher Paolo Freire, focuses on empowerment and social justice to reduce inequities. (27) Its principles include acknowledging participants as active in their own learning process and using their lived experience to build knowledge and take action. In this approach, the goal of training and education is for participants to be active change agents in their communities to resolve problems and improve their lives. This approach has increasingly been adopted into health promotion programs and emerging evidence has demonstrated its effectiveness in improving health outcomes (28, 29).

The C3 Project developed a list of 10 core CHW roles and 11 core skills to describe the scope and practice of CHWs, including in the health care team. (29) Although the C3 Project was not focused specifically on HIV care, the roles and skills provide a framework for developing the CHW's role. The training curricula was informed by these core competencies and were mapped specifically for the role of the CHW in the care team in supporting a person with achieving each outcome along the HIV care continuum: linkage to care, retention in care, adherence to treatment and viral suppression (30).

A team of nine experts in HIV, education, training, supervision and practice developed and implemented both curricula. By profession, the team represented two professional trainers for state CHW training programs, two professional trainers and organizational experts in HIV for clinicians and non-clinicians, two CHW supervisors working in HIV clinical care settings and two CHWs currently working as part of HIV health care teams for city or county health departments. The director of the team was an University faculty member with substantial experience in HIV and peer and CHW training. Three members of the team were persons with HIV. Once the curricula were developed, a team of outside experts reviewed and provided feedback before implementation. This seven person review team consisted of one HIV primary care physician, three CHW and HIV program directors and three CHWs working non-HIV chronic health conditions.

The curricula included 80 h for CHWs and 20 h for supervisors. Tables 1, 2 describe the topics and hours for each curriculum. In line with the principles of Popular Education, the training modules were designed to be learner-centered with content and activities designed to engage in discussion and address the structural inequities contributing to the HIV epidemic, such as racial, gender, sexual orientation and economic disparities. CHW participants engaged in approximately 16 h of HIV-based content to educate and support persons with HIV. This included HIV basics; the HIV life cycle and its treatment; managing stigma and disclosure associated with HIV; gender identity, sexual orientation and other factors that contribute to discrimination; and the impact of living with a mental health or substance use disorder on HIV treatment adherence. CHW participants also received training on strategies to support treatment adherence and harm reduction to promote healthy behaviors. In addition to the HIV knowledge base, CHWs received 64 h of training focused on CHW core competencies and skills, including communication, interpersonal relationship-building, service coordination and navigation, capacity building, advocacy, education and facilitation, individual and community assessment, outreach, professional skills and conduct, and evaluation and research skills. The training program was augmented with specialty topics such as addressing intimate partner violence, supporting clients in crisis and a trauma informed care course.

**Table 1:** HIV core topics and hours completed by CHWs and supervisors.

**Training topics**	**Time (min) o training**	**C3 Core competency**	**Participants**
Introduction to social determinants of health	15	Knowledge base	CHWs
Introduction to community health work in the context of the HIV care continuum	15	Knowledge base	CHWs
HIV treatment cascade/care continuum	15	Knowledge base	CHWs
HIV 101	45	Knowledge base	CHWs
HIV viral life cycle	60	Knowledge base	CHWs
Viral cycle: medication	60	Knowledge base	CHWs
Adherence, labs, resistance	90	Knowledge base	CHWs
PEP, PrEP, and TASP	40	Knowledge base	CHWs
Multidisciplinary care teams: how a CHW fits Into an HIV care team	30	Knowledge base	CHWS & supervisors
Stigma and discrimination	60	Knowledge base	CHWs
HIV and other co-morbidities	60	Knowledge base	CHWs
HIV 102: sexual health	45	Knowledge base	CHWs
Addressing medication adherence and treatment parts 1 & 2	120	Knowledge base	CHWs & supervisors
Promoting medication adherence	75	Knowledge base	CHWs
Support for managing HIV disclosure	60	Knowledge base	CHWs & supervisors
Sexual health let's talk about sexuality	75	Knowledge base	CHWs
Supporting clients with disclosure	75	Knowledge base	CHWs
Intimate partner violence^*^	90	Knowledge base	CHWs
Medications & drug interactions^*^	60	Knowledge base	CHWs
HIV and aging^*^	60	Knowledge base	CHWs
HIV and substance use^*^	60	Knowledge base	CHWs

* *Denotes a Virtual session. All other session were in-person*.

**Table 2:** CHW core competencies and skills and hours completed by CHWs and supervisors.

**Topics and session**	**Training hours**	**Core competencies (C3)**	**Participants**
Background and information about training	20	Providing culturally appropriate health education information	CHWs & supervisors
Trust building	30	Providing culturally appropriate health education information	CHWs & supervisors
Introduction to popular education	40	Providing culturally appropriate health education information	CHWs & supervisors
Who are community health workers? History, roles, skills, and qualities	80	Providing culturally appropriate health education information	CHWs & supervisors
What does it mean to be a CHW supervisor	90		Supervisor
Defining a multi-disciplinary team	30		Supervisor
Communicating with staff and CHWs	60		Supervisor
Orienting team members: advocacy for CHWs as part of the care team	35		Supervisor
Supervision frequency, types, modes	70		Supervisor
Providing feedback and mentoring	60		Supervisor
Trauma Informed supervision	60		Supervisor
Establishing and supporting boundaries and confidentiality	75		Supervisor
Recognizing transference and countertransference	45		Supervisor
Establishing and promoting the importance of self care	30		Supervisor
CHW and multidisciplinary teams	30		Supervisor
Explaining CHW role to multi-disciplinary care teams	30		Supervisor
Challenges and solutions to working on a team	45		Supervisor
Prioritizing and organizing your time as a CHW	60	Care Coordination, Case Management, System Navigation	CHWs & Supervisors
Patient navigation^*^	60	Care coordination, case management, system navigation	CHWs & Supervisors
Documentation skills^*^	40	Care coordination, case management, system navigation	CHWs & Supervisors
Care planning^*^	60	Care coordination, case management, system navigation	CHWs & Supervisors
Case conferencing on multi-disciplinary teams	60	Care coordination, case management, system navigation	CHWs & Supervisors
Successes and barriers to collaboration	30	Care coordination, case management, system navigation	CHWs & Supervisors
Appropriate and workable boundaries	30	Professional skills & conduct	CHWs & Supervisors
Professional boundaries & ethics^*^	60	Professional skills & conduct	CHWs & Supervisors
Boundaries & confidentiality II	90	Professional Skills & Conduct	CHWs & Supervisors
Building a community network ^*^	30	Advocating for individuals & communities	CHWs & Supervisors
Evaluation overview of project	45	Participating in evaluation and research	CHWs & Supervisors
Community based participatory research & assessment^*^	90	Participating in evaluation and research	CHWs & Superivsors
Wrap up reflections & self-care	90		CHWs & Supervisors
Crisis intervention & prevention	210	Providing coaching and social support	CHWs & Supervisors
Harm reduction	120	Providing coaching and social support	CHWs & Supervisors
Motivational interviewing II	60	Providing coaching and social support	CHWs & Supervisors
Medication adherence II	60	Providing coaching and social support	CHWs & Supervisors
Outreach to engage and retain hard to reach populations^*^	120	Conducting outreach	CHWs & Supervisors
Protecting personal safety	20	Conducting outreach	CHWs & Supervisors
Medication and treatment II	60	Providing coaching and social support	CHWs & Supervisors
Motivational Interviewing and active listening practice	60	Providing coaching and social support	CHWs & Supervisors
Understanding empathy through the lens of cultural humility	60	Cultural Mediation among individuals, communities and health service systems	CHWs & Supervisors
Trauma-informed care	180	Cultural Mediation among individuals, communities and health service systems	CHWs & Supervisors
Strengths-based feedback	105		Supervisor
Managing conflict and difficult conversations	60		Supervisor
Open forum	75		Supervisor
Guided movement -self-care techniques	15		Supervisor
Self-care/stress management techniques for self and team	80		Supervisor
Practicing self-care techniques	25		Supervisor
Popular education principles: education and facilitation skills	60	Providing culturally appropriate health education information	CHWs & Supervisors
Popular education methods and workshop preparation	60	Providing culturally appropriate health education information	CHWs & Supervisors
Use of public health concepts and approaches^*^	120	Providing culturally appropriate health education information	
Leading groups and facilitation skills	60	Providing coaching & social support	CHWs & Supervisors
Facilitation challenges	45	Providing coaching & social support	CHWs & Supervisors
Advocacy skills	60	Advocating for individuals & communities	CHWs & Supervisors
Formal avenues of advocacy	60	Advocating for individuals & communities	CHWs & Supervisors
Empowering leadership	90	Building individual and community capacity	CHWs & Supervisors
Team building skills to support integration	60	Care coordination, case management, system navigation	CHWs & Supervisors
Communication as part of a team	90	Cultural mediation among individuals, communities and health service systems	CHWs & Supervisors

* *Denotes a Virtual session. All other sessions were in person*.

The supervisor curriculum included training on administrative and clinical supervision for CHWs and care team members, mentoring and providing feedback, conflict resolution and orienting health care team members to the role of the CHW. Supervisors were also invited to participate in HIV training sessions to learn strategies and techniques for promoting treatment adherence and support with stigma and disclosure. Both CHWs and supervisors received training on the role of the CHW in the health care team and strategies for working as part of an interdisciplinary team. Cultural humility and trauma informed care were also key modules for both the CHWs and supervisors. Each training session also incorporated “dinamicas” (movement exercises) and self -care techniques, usually 30 min activities, which included yoga or use of affirmations with the group that CHWs could use for themselves or with clients as a way of coping and managing the stress in their lives due to their HIV status.

## Learning Environment and Training Modality

The training curricula were delivered over the course of 1.5 years from September 2017-January 2019 via in-person and virtual sessions. The initial core training (September 2017) included 5 days (~40 h) for CHWs and 2 days (approximately 16 h) for supervisors. The initial program focused on the HIV core content, the CHW role in care teams, working in teams effectively, motivational interviewing skills, cultural humility and trauma informed care. CHWs and supervisors participated in sessions both together and separately as noted in Tables 1, 2. Following the initial program, a detailed schedule and courses were mapped to deliver content via 1–3 day in-person sessions (December, 2017, April and July 2018, January 2019). In between the in-person sessions, virtual training sessions were conducted for approximately 60–90 min in length to complete the 80-h program for CHWs. Supervisors were invited to attend virtual sessions. The training team mapped training content to modality (virtual vs. in-person), prioritizing topics for interactive dialogue and group skills (motivational interviewing skills to promote treatment adherence, trauma informed care, facilitation skills) for in-person and subject matters such as assessment, evaluation and research and content (IPV) for virtual sessions. The in-person training sessions were conducted in conjunction with “Learning Collaborative Sessions” which focused on organizational capacity building and opportunities for the 10 sites to share innovations and challenges with integrating CHWs into the care teams and agencies. The lessons from the learning collaborative sessions are detailed in a final implementation manual: https://targethiv.org/chw (31). All participants were awarded certificates of completion based on the number of training sessions completed.

The training team met monthly to discuss training content, logistics and plan for modifications and adjustments to the sessions. After each in-person session, the training team debriefed to discuss adjustments to the agenda, groups dynamics and strategies to improve the learning environment. Experienced trainers in adult learning principles and Popular Education paired with new CHW trainers and/or HIV content experts to enhance activities and encourage a participatory and learner-centered approach.

## Training Evaluation Methodology

A mixed-methods evaluation was designed to assess the impact of the training on CHW and supervisor skills and confidence in applying those skills. All in-person sessions used a pre-posttest design with follow up assessments completed at 1 and 3 months post-training. The assessment consisted of 19 items across three domains: (1) HIV-related content including educating about the viral life cycle, knowledge of treatment and medication side effects and supporting clients with stigma and disclosure; (2) competency in using motivational interviewing and trauma-informed care techniques and (3) tasks and responsibilities as a member of the health care team including communicating with providers about clients, boundaries and self-care. Each item was scored on a 5-point Likert scale, with 1 = low confidence and 5 = high confidence. In-person and virtual trainings consisted of a qualitative methodology of four questions immediately post-training. The four questions were: (1) What information covered today were most impactful/helpful? (2) What information do you wish we covered? (3) What three things worked well today? (4) What three things could be improved?

Descriptive analysis was used to assess trends in changes in CHW confidence over time from the pre-, post-test survey. Thematic content analysis was used to describe the impact on participant knowledge and application to their role as a CHW or supervisor, and for lessons learned for continuing education.

## Results

The program trained a total of 23 CHWs and 22 Supervisors across the 10 sites. The majority of the CHWs were female (16), Black (17) or Hispanic (3) or other multiracial group (3) and had some college or post-secondary education (20). While data on participant HIV status was not formally collected as part of the evaluation, nine participants voluntarily disclosed that they were a person living with HIV during the project. Most had experience working in HIV and 45% were new to working in the CHW role. Supervisors were predominantly female, and identified as Asian (1), Black (15), Hispanic (2) or other non-white identity (1) and had graduate degrees (15). While the majority had been in a supervisory role for >3 years, fewer reported experience in the field of HIV. Table 3 describes CHW and supervisor characteristics.

**Table 3:** CHW and supervisor characteristics (*n* = 45).

	**Community health workers**	**Supervisors**
	**(*n* = 23)**	**(*n* = 22)**
Gender		
Male	7 (30%)	1 (3%)
Female	16 (70%)	21 (97%)
Race/ethnicity		
Asian	0 (0%)	1 (4.5%)
Black/African-AmericanHispanic/Latinx	17 (74%) 3 (13%)	15 (68%)2 (9.0%)
Other	2 (10%)	1 (4.5%)
Multiracial (Black-Native American)	1 (3%)	0 (0%)
White	0 (0%)	3 (14%)
Primary language		
English	20 (87%)	22 (100%)
Spanish	3 (13%)	0 (0%)
Primary education		
High school/GED	2 (9%)	0 (0%)
Some college/post college	14 (61%)	4 (17%)
College graduate	3 (13%)	3 (13%)
Graduate school/professional school	3 (13%)	15 (70%)
Other	1 (4%)	0 (0%)
Years in position (mean, range)	2.6 (1–5)	
Years at the organization (mean, range)	3.0 (1.5)	
Years working in HIV	<3 year 3 (15%)	<3 years 7 (31%)
	More than 3 years 20 (85%)	3 or more years 15 (69%)
Years of experience in role CHW/supervisor (mean, range)	2.9 (1–6)	<3 years 3 (15%)
	New (<1 year) 10 (45%)	3 or more years 19 (85%)

Twelve CHWs completed the 80-h training program; 11 did not complete it. The range of hours completed was 23–80 h. The most common reason for lack of completion was due to CHWs leaving their agency for other positions or educational opportunities. Three supervisors completed the 20-h supervisor program and 38 h of additional sessions offered through the CHW 80 curricula. The range across supervisors was 7–58 h of training. The most common reason for lack of completion was competing clinic responsibilities and challenges with traveling to attend in-person sessions.

### Changes in CHW and Supervisor Skills and Confidence as Part of the HIV Care Team

Post-training results found improvements in self-reported general HIV knowledge among the CHWs and educating clients specifically about HIV treatment and viral suppression. Greatest improvement was reported related to providing trauma-informed care to clients (mean 3.7–4.5). Confidence in communicating needs to administrative and supportive supervisors decreased slightly in the post period (4.8–4.7). Overall participants reported high confidence in applying information from the training in their daily work in the post period (Tables 4A,B).

**Table 4A:** Changes in CHW confidence from initial 40-h HIV training (mean, SD).

**Item 1 = low, 5 = high**	**Pre-test**	**1 Month post-test**	**3 Months post-test**
	***N* = 11**	***N* = 8**	***N* = 9**
General HIV/AIDS knowledge^*^	4.45, 0.69	4.88, 0.35	4.56, 0.73
Comfort explaining the HIV virus to a participant	4.64, 0.92	4.88, 0.35	4.78, 0.44
Confidence in your ability to explain the importance of medication adherence and viral suppression to your participants	4.73, 0.65	5, 0	4.89, 0.33
Understanding of your roles as a CHW within the HIV care team	4.27, 1.79	5, 0	5.11, 1.54
Confidence in your ability to explain how your role as a CHW impacts the HIV care continuum^*^	4.27, 1.79	4.88, 0.35	4.44, 0.53
Understanding of PrEP, PEP, and Treatment as Prevention (TasP) and who can benefit from it^*^	4.09, 0.54	4.88, 0.35	4.67, 0.50
Confidence in understanding your role and tasks as a member of the HIV care team	4.27, 0.79	5, 0	4.22, 0.83
Confidence in your ability to provide trauma informed care to participants^*^	3.73, 0.79	4.5, 0.76	4.33, 86.7
Confidence in your ability to use motivational interviewing techniques with participants	4.18, 0.75	5, 0	4.33, 0.71
Confidence in your ability to manage your time as a CHW, including your case load	4.45, 1.63	5, 0	4.89, 1.69
Confidence to document your work with participants in the electronic health record^*^	4.18, 1.40	4.88, 0.35	4.89, 0.33
Confidence in your ability to communicate about participants with other members of the HIV care team in your organization	4.81, 0.40	4.88, 0.35	4.67, 0.50
Comfort communicating your needs to your administrative and supportive supervisors	4.81, 0.40	4.75, 0.71	4.22, 1.09
Confidence in your ability to maintain appropriate boundaries with participants	4.64, 0.67	4.88, 0.35	4.44, 0.53
Confidence in your ability to create time and space for self-care throughout your work	4.55, 0.69	4.75, 0.71	3.89, 1.05
Skills creating partnerships with other community members and stakeholders	4.81, 0.40	5, 0	4.67, 0.50
Comfort in helping participants to address and manage stigma and disclosure related to HIV or other conditions	4.73, 0.47	5, 0	4.44, 0.73
Overall satisfaction with the CHW training	-	5, 0	4.78, 0.44
Confidence in your ability to apply what you learned in the CHW training at work	-	5, 0	4.89, 0.33

* *statistically significant*.

**Table 4B:** Changes in supervisor confidence from initial 16-hour training (mean, SD).

**Item 1 = low, 5 = high**	**Pre-test**	**1 month post-test**	**3 months post-test**
	***N* = 12**	***N* = 9**	***N* = 5**
General HIV/AIDS knowledge	4.83, 0.39	4.78, 0.44	4.60, 0.55
Comfort identifying and addressing stigmatizing behavior in the HIV care setting	4.75, 0.45	4.56, 0.53	4.80, 0.45
Confidence in describing CHW roles and skills	4.17, 0.84	4.45, 0.53	4.60, 0.54
Confidence in your ability to describe the CHW role on the HIV care team within your organization	4.25, 0.87	4.45, 0.53	4.60, 0.54
Confidence in your general ability to adequately supervise a CHW who is part of the HIV care team	4.5, 0.80	4.56, 0.53	4.60, 0.54
Confidence in your ability to provide trauma informed supervision	3.67, 0.78	3.78, 0.67	3.80, 0.45
Confidence in your ability to promote self-care for the CHWs you supervise	4.50, 0.52	4.56, 0.53	4.80, 0.45
Confidence in your ability to maintain appropriate boundaries with CHWs you supervise	4.67, 0.49	4.67, 0.50	5.00, 0
Confidence in your ability to provide feedback to your CHWs you supervise	4.67, 0.49	4.78, 0.44	4.80, 0.45
Confidence in your ability to recognize countertransference in yourself during CHW supervision	4.00, 0.85	4.45, 0.73	4.40, 0.55
Confidence in your ability to recognize transference in a CHW during supervision	4.00, 0.85	4.45, 0.73	4.20, 0.55
Confidence in your ability to advocate for CHWs on your HIV care team	4.67, 0.49	5.00, 0.00	4.08, 0.45
Overall satisfaction with the CHW supervisor training	-	4.67, 0.50	4.80, 0.45
Confidence in your ability to apply what you learned in the CHW supervisor training at work	-	4.78, 0.44	4.80, 0.45

Similarly, supervisors reported improvements in understanding HIV topics and being able to support CHWs in their work with clients. Confidence in providing-trauma-informed supervision had the lowest mean score although it improved over time (3.67–3.8). Supervisors also reported increased confidence in their ability to support and advocate for CHWs as part of the care team (Tables 4A,B).

### Impact on Knowledge and Skills to Strengthen Care for People With HIV

Three key themes that emerged from the open-ended questions in the post-training evaluations: (1) new information and content related to HIV, (2) new techniques and skills to work with clients on HIV or during supervision, and (3) recommendations to the training team related to training modalities for the HIV workforce. Below we describe the most frequently reported themes from these areas and examples of narrative quotes from CHW and supervisors.

***Learning and applying HIV content in their work with clients*:** CHW participants described the importance of learning new information about HIV and the viral life cycle, for whom and how HIV medications and PrEP work, and treatment as prevention. CHWs also mentioned the usefulness of how to look up resources and tailor information for their clients. One CHW reported:

“*I used the knowledge of medications of HIV by looking up the chart and checking medications and also learning what works for some people may not work for others as far as different medications.”*

Other CHWs reported they used the exercises on how to explain and educate clients about HIV. As one participant described: “*…For me utilizing lab results to explain to clients effectively a better understanding of the stages of HIV, the life cycle of HIV and what it all means.”*

CHWs and supervisors also found it useful to learn about the HIV care continuum and share this information with clients. While many CHWs and supervisors were familiar with each stage, learning to assess where clients were in the care continuum and potential challenges were essential to helping clients with self-efficacy to enter care, treatment adherence and ultimately in reaching viral suppression. The training also provided motivational techniques and affirmations to clients for continued success.

***New techniques and skills to work with clients and during supervision:****One* of the most frequently mentioned new skills for CHWs was on *documentation and care planning* for clients as part of the HIV care team. CHW participants described the importance of learning how to document their work with clients in charts and differentiating between information that might be mandatory for health records vs. what is unnecessary to protect a client's privacy. As one CHW reported*… “I appreciated the tips on how to write a specific note using the SOAP technique. This gave me example of how I can keep a record of what we did with a client.”*

***Implementing Motivational Interviewing and self-care skills:***Using motivational interviewing (MI) skills such as active listening and avoiding assumptions were reported as useful skills in working with clients in the post training period. A CHW reported that MI helped them learn how to effectively transition clients to another team member and also how to manage client: “*I used to take resistance personal but I don't do that anymore… I learned to be able to handle tough situations with patients, i.e., when they aren't responding to your questions right in front of you.”*

Self–care techniques were often most frequently mentioned as useful and applied in CHWs' daily work for themselves or clients. As one CHW reported: “*Self-reflection has been a major part of my life since this training, also MI,”* and another described: “*my self-care has improved by turning the work cell phone completely off after business hours…*”

Supervisors similarly reported being able to implement self-care techniques for their staff and using reflective communication as a tool to help CHWs manage their work with clients and the stress in their lives.

***Trauma-informed care and supervision:***Both CHWs and supervisors frequently reported the importance of and continued training and support on trauma-informed care techniques. In the initial training, few participants had heard of this approach and its importance in working with clients with HIV.

***CHW scope of practice within the care team:***Supervisors were able to apply techniques from the trainings to provide encouragement and feedback to CHWs and support a healthy CHW-client relationship. Supervisors reported learning communication techniques to support CHWs as valued members of the team and ensure integration into the care team. One supervisor reported “*The training helped me to tailor my supervisory approach to the needs of the CHW which are different from my existing staff who do clinical work (and) are mostly trained mental health professionals.”* Another reported: “*I was able to use my knowledge and understanding of the CHW role to inform other workforce members and community partners (about the role) while at the same time providing support to my CHW*.” From a CHW perspective, one participant described the training as “…*helpful in providing valuable insight and practice on preparing and addressing resistance about the CHW role from other members of the team*.”

***Recommendations on the training modality:***In addition to new information learned and the application of skills, themes also emerged about the delivery of content for the HIV workforce. Both CHW and supervisors reported the *usefulness of individual and joint sessions between CHWs and supervisors*. CHWs reported the individual sessions were important to have time to talk with other CHWs about ways to effectively communicate needs with their supervisors. For supervisors, this was the first time receiving training and being in a group with other colleagues to talk about supervision techniques. They described the value of training in learning from their peers on how to provide direct, honest feedback and coaching with supervisees to help move them forward with their relationships with clients and other team members. The joint CHW-supervisor sessions then allowed participants to practice communication skills as a team, to have a dialogue around CHW integration challenges, and find resolutions together in a safe environment with others experiencing similar challenges. Finally, the supervisors and CHW participants appreciated the interactive nature of the training sessions which allowed participants to share current client cases, use and demonstrate self-care techniques, and role play scenarios so participants could “*see in real time how problems can be addressed…”*.

## Discussion

There is increasing need for evidence-based training tools and materials to strengthen CHW HIV workforce both in US and international settings. UNAIDS declared a need to train 2 million CHWs in Africa as part of the plan for ending AIDS and ensuring sustainable health for all across the continent. In order to scale up and reach this goal, the agency calls for the widespread use and adaptation of training resources to support the CHW workforce and other health care workers to minimize professional resistance. (32) In the US, the National HIV/AIDS Strategy 2021–2025 explicitly calls for the use of CHWs, including peer navigators, as part of the public health workforce and health care delivery system to effectively identify, diagnose, and provide holistic care and treatment for people with HIV to increase viral suppression rates. (2) Our curricula contribute to this body of knowledge providing both HIV content and other core CHW competencies and skills, such as cultural mediation, service navigation and coordination, individual and community assessment and educational support for people with HIV. A unique aspect of our model was including training for CHW supervisors that was specific to an HIV care environment.

In addition, the article presents a participatory process and implementation of comprehensive CHW and supervisor training curricula and program for integrating CHWs into the HIV care team to improve care and treatment access for newly diagnosed and out of care people with HIV. The curricula were developed and delivered by a team of CHWs and supervisors working in HIV care and professionals in training and organizational development. This interdisciplinary team helped to create a practical learning experience with real world examples to build the capacity and skill of CHWs and supervisors working with clients with complex morbidities and competing medical and social needs. The curricula used innovative methods grounded in Popular Education to create both in-person and virtual sessions focused on HIV and CHW core competency skills. A recent systematic review of CHW training programs in African–American and Latinx communities found a continued need for creating and disseminating training programs grounded in evidence-based approaches to engage these communities and rigorous evaluation designs that directly link CHW training programs to health outcomes. (33) The development and approach of these curricula focused on and included people with HIV from racial/ethnic minority communities. The results from this training program contribute to the body of evidence on CHW competencies in both primary health care and HIV specialty care and may be replicated for other chronic disease conditions.

There is limited published information about CHW and supervisor curricula and their impact on CHW skills and team integration. (7, 16, 32, 34) One unique contribution of this work is the theoretical framework and approach. Grounded in the Popular Education model, the curricula provides a framework to deliver content that is learner-based and driven. HIV content was developed and adapted by CHWs and supervisors working in the field and included persons with lived experience with HIV. Cases and problem-solving skills were developed using real life scenarios that CHWs and supervisors were encountering in their practice. This content often included persons with HIV dealing with major barriers to accessing care including stigma related to HIV, sexual orientation, race, and gender identity. Interactive training sessions were provided to encourage dialogue among participants on how to resolve client challenges that would promote linkage and retention in care, adherence to treatment and viral suppression. Results from this analysis indicate that training participants, including those who were experienced in community health work, gained new information that could be relevant and useful in the field.

Another key feature of the curricula was its approach in dismantling silos and enhancing conversation to address power dynamics between CHWs and supervisors and the health care team and people with HIV. The use of joint and individual sessions allowed dialogue between and across roles that is essential in health care teams, which often is hierarchical. Our findings highlight the importance of structured trainings that focus on teamwork skills, highlighting the roles that each team member, specifically CHWs, play in supporting care and treatment adherence. Role playing scenarios, where CHWs can practice educating and explaining in appropriate lay terms how HIV affects a person's immune system and how medications work, was important to new CHWs in learning the role they can play in HIV care. CHWs are well poised to discuss not only the biological dimensions of HIV, but to help clients employ strategies that address their social needs that often impact treatment adherence. These curricula included sessions on communicating effectively in sharing pertinent information with care team members and building client communication skills with health care providers. Having CHWs and supervisors as part of the training team was essential to creating a learner-centered environment and sharing problem-solving techniques to address challenges with the health care system that people with HIV may experience.

The evaluation findings also signal the importance of training members of the care team on the scope and practice of the CHWs. Our curricula include approximately 8 h of joint sessions with CHWs and supervisors on the CHW role, working as an interdisciplinary team and addressing challenges as a team. Training approaches and methodologies that incorporate opportunities to integrate health care team members and learn about each other's role, unique skills and perspectives are necessary to address economic, social and medical challenges that may hinder viral suppression for people with HIV. Both CHW and supervisors reported the training format and curricula provided an opportunity to network and learn from other peers across the country (35).

Another lesson learned was the role of continuing education on motivational interviewing and trauma-informed practice techniques for CHWs and supervisors. CHWs reported their use of these techniques with clients to address adherence challenges. Self-care techniques, modeling and affirmations used during the training were also replicated in their work with clients.

Virtual training was also feasible to implement but more effective if CHW participants had a webcam and could interact more readily in the group. Our trainings program was implemented prior to the COVID-19 pandemic and given that many health clinics have invested in technology and supported staff to deliver client services via telehealth, CHWs and supervisors may now have greater comfort with remote learning in a post pandemic world. Virtual trainings that engaged participants with brief exercises that practiced skills, provided information on how to look for resources for the clients and engaged group in feedback were more highly received. Specific content areas delivered by experts such as learning about Intimate Partner Violence or sharing tips on documenting information from clients were also effectively delivered in a virtual format.

Our CHW curriculum is not a substitution for certification programs offered in many states for CHWs. However, the 16-h HIV curricula could serve as specialty area training or for CHW continuing education. This was one reason that the CHW curriculum was informed by state certification programs, such as those in Oregon and Texas, and built on the C3 Project core competencies for CHWs.

Finally, a key lesson in designing these curricula was engaging CHWs in the cross-sharing of their varied experiences across the country. The opportunities to share resources, present client challenges for discussion and generate solutions from within the group were critical to a productive learning environment. Approximately 50% of the original cohort completed all 80 h of the curricula, showing the benefit of the training methodology. This training approach, which focused on HIV-specific content with core competency training, could serve as a model for CHWs working in primary care settings and with populations experiencing multiple chronic health conditions and social needs.

### Strengths and Limitations of Project

Our training program was developed with a cohort of HIV primary clinics who recognized the need and value to have a CHW as part of the care team. The ten sites had applied for and received funding to support the program. All the programs were Ryan White funded clinics, which have a long history of involving people with HIV in their service delivery as part of this mission and culture. The ten sites represented diverse organizational settings, including federally qualified community health centers and outpatient clinics affiliated with hospital systems. However, our results may not be generalizable and readily replicable to all health care settings, especially those with no previous CHW experience.

In addition, given our small sample size and follow up rate, further study is needed examine the curricula impact on CHW and supervisor knowledge, skills and confidence. The level of training was also supplemented by a learning collaborative and coaching sessions to strengthen collective identities and supportive mechanisms. CHWs met monthly as an affinity group while supervisors met quarterly. Coaching sessions were provided monthly to each of the 10 Ryan White HIV/AIDS Program sites. Coaching sessions were participant-driven, with topics coming from the site staff and facilitated by an organizational training expert. Some example of topics that were discussed included technical assistance needs, program successes, the CHW role, new partnerships to serve the community, and any trainings external to this project that were attended by CHW program staff. This additional time beyond training may have contributed to the ability of CHWs to more readily integrate into the health care team. It was beyond the scope of this evaluation to tease out the effects of the contributions between training and coaching sessions. Future implementation research may assess these strategies separately to elucidate the contributions toward effective CHW integration in the HIV care team.

Finally, ~50% of the cohort of CHWs and the majority of supervisors completed the entire training program. Creating incentives for both CHWs and supervisors to complete training sessions such as providing continuing education credit, half-day in-person sessions and scheduling virtual sessions during non-clinic hours or days may have improved participation and course completion. Our training curricula promoted professional development and skills such as advocacy skills and empowering leadership. In our supervision curriculum, discussions regarding CHW professional development were also encouraged as part of administrative and clinical supervision. However, the reality of finding opportunities for CHWs to move up within their organizations could be challenging, given funding and agency policies. Another factor contributing ability to complete the full training curricula may be due to the modality of the training. Many of our in-person training were one-day sessions conducted in Boston and occurring just prior to a 2-day learning collaborative to explore organizational integration. For some participants, primarily supervisors, it was challenging to be out of the office and travel out of town for three full days due to competing responsibilities. Thus, some staff elected to miss the training session.

### Future Directions for Practice and Research

This training curricula and program contributes to the body of evidence of the training process and strategies for CHWs and supervisors working in primary health care settings. One of the key successes of the curricula was joint sessions between CHWs and supervisors, which enhanced dialogue about promoting client knowledge and decisions about HIV adherence and treatment, as well as built trust and supportive relationships between care team members. Establishing similar training strategies can improve the quality of care by reducing duplication in services and encouraging clearer scopes of practice between CHWs and other team members. Finally, a further study could also look at the impact of the training on client outcomes such as retention in care.

These CHW and supervisor curricula also highlight the importance of including members on the training team who are CHWs and share gender, race/ethnicity and lived experience. The experiential learning approach grounded in Popular Education led to improved knowledge, skills, and confidence of CHWs and supervisors. Future studies could examine the impact of dose and types of sessions on HIV and other health outcomes.

## Conclusion

The interactive participatory practice-based curricula allowed supervisors and CHWs to share experiences from the workplace and solicit input from peers for problem resolution and implementation of new policies and practices. This training approach focused on HIV-specific content with core competency training, and could serve as a model for CHWs working in primary care settings with populations experiencing multiple chronic health conditions and social needs.

## Data Availability Statement

The raw data supporting the conclusions of this article will be made available by the authors, without undue reservation.

## Author Contributions

SR, LSM, MS, and AB contributed to the conception and design of the study. SR wrote the first draft of the manuscript. LSM, AB, and MS conducted data analysis. BP, JD, AD, SP, LM, PJ, EE, M-LD, and SB contributed to section of the manuscript. All authors contributed to the manuscript revision, read and approved the submitted version.

## Conflict of Interest

The authors declare that the research was conducted in the absence of any commercial or financial relationships that could be construed as a potential conflict of interest.

## Publisher's Note

All claims expressed in this article are solely those of the authors and do not necessarily represent those of their affiliated organizations, or those of the publisher, the editors and the reviewers. Any product that may be evaluated in this article, or claim that may be made by its manufacturer, is not guaranteed or endorsed by the publisher.
